# Supplementary material for: A conserved odorant binding protein is required for essential amino acid detection in Drosophila
Source: Commun Biol. 2019 Nov 22;2:425. doi: 10.1038/s42003-019-0673-2 (PMC6874667; doi:10.1038/s42003-019-0673-2)
Supplement: Supplementary file 1 — Supplementary Information [file 42003_2019_673_MOESM1_ESM.pdf]

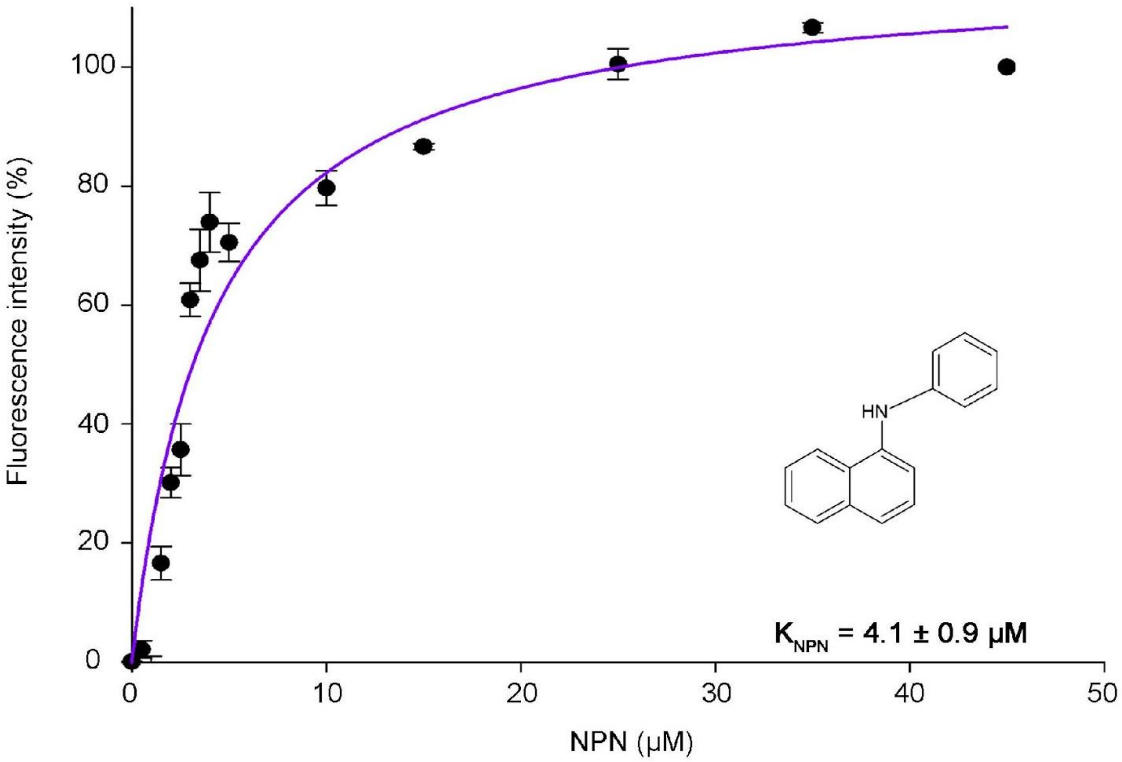

Supplementary Figure 1. Binding of NPN to OBP19b. Curve of titration of OBP19b by NPN. The NPN dissociation constant was measured by titration of a 2 µM OBP19b with aliquots of 10 mM NPN to a final concentration of 0.5-50 µM. The excitation wavelength of NPN was 337 nm, and the emission wavelength was 415 nm.

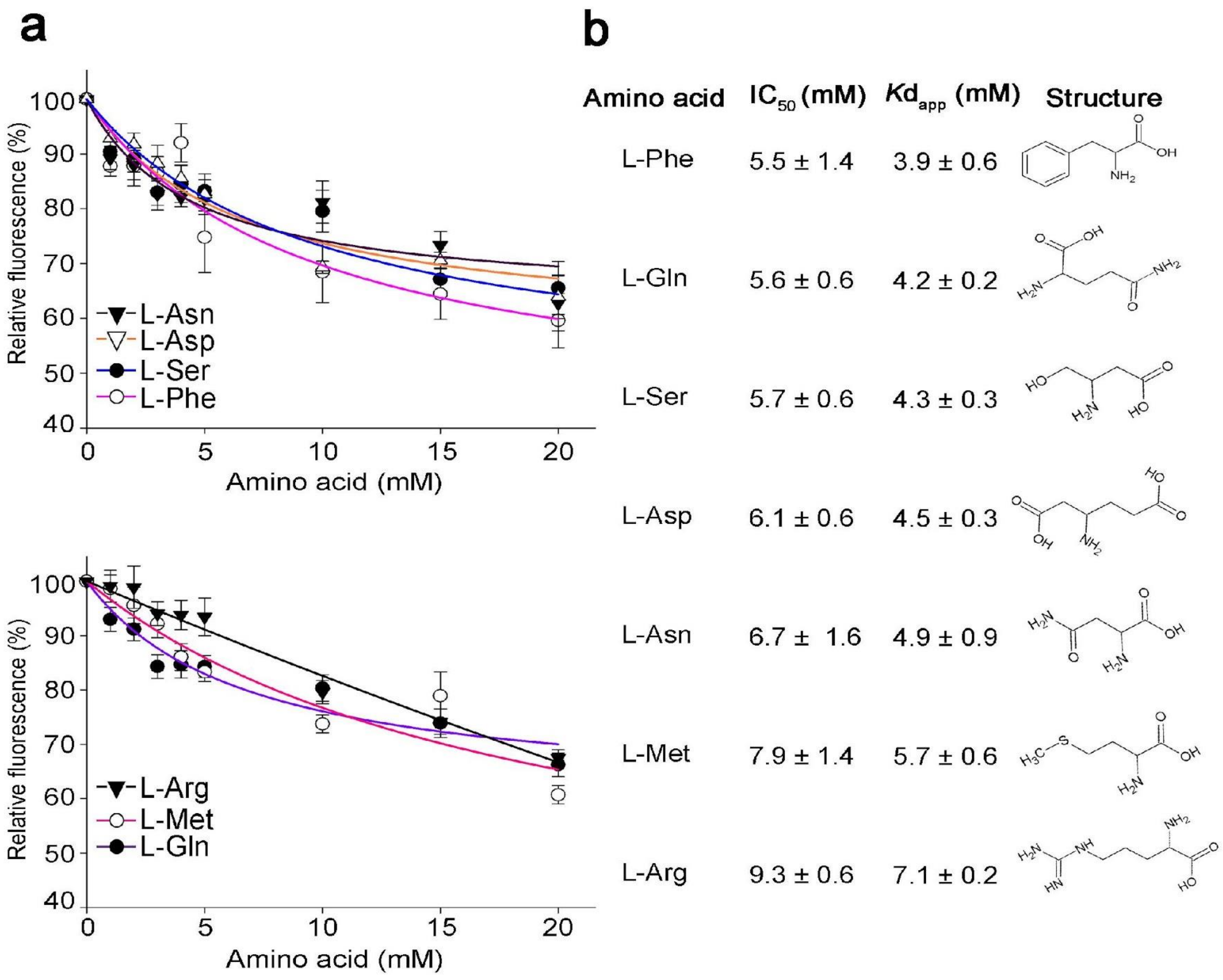

Supplementary Figure 2. OBP19b binding affinity for amino acids. (**a)** Fluorescent competitive binding curves of OBP19b bound to L-phenylalanine (L-Phe), L-asparagine (L-Asn), L-aspartic acid (L-Asp), L-serine (L-Ser), L-glutamine (L-Gln), L-methionine (L-Met) and L-arginine (L-Arg). (**b)** The concentration of ligands multiplied by one-half of the initial fluorescence value of NPN (IC_{50}) and the dissociation constant (Kd_{app}) of OBP19b ligands with the respective structure of each. Data values represent the mean ± SEM. N=4-8.

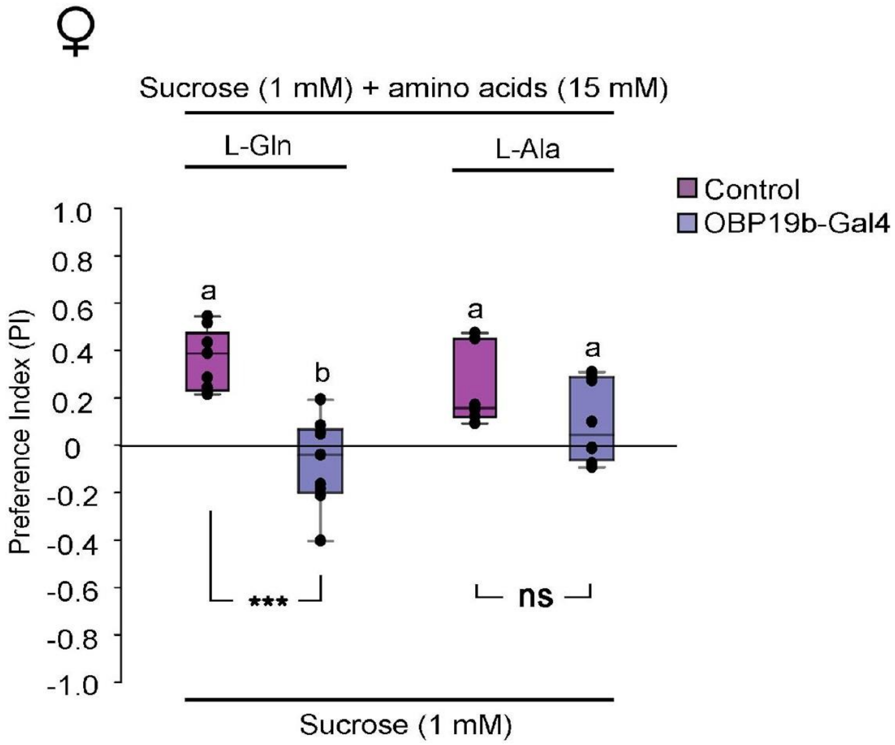

Supplementary Figure 3. Amino acid taste preference in mated female flies. Preference for L-glutamine (L-Gln) and L-alanine (L-Ala) by flies of control and mutant OBP19b1 genotypes. Both control and amino acid-rich solutions contained 1 mM sucrose. The amino acid solution contained 15 mM L-Gln or L-Ala. The letters indicate significant differences determined by the Kruskal Wallis test and post hoc Wilcoxon test: ns=nonsignificant; * p<0.05;** p<0.01; *** p<0.001). N=8-11.

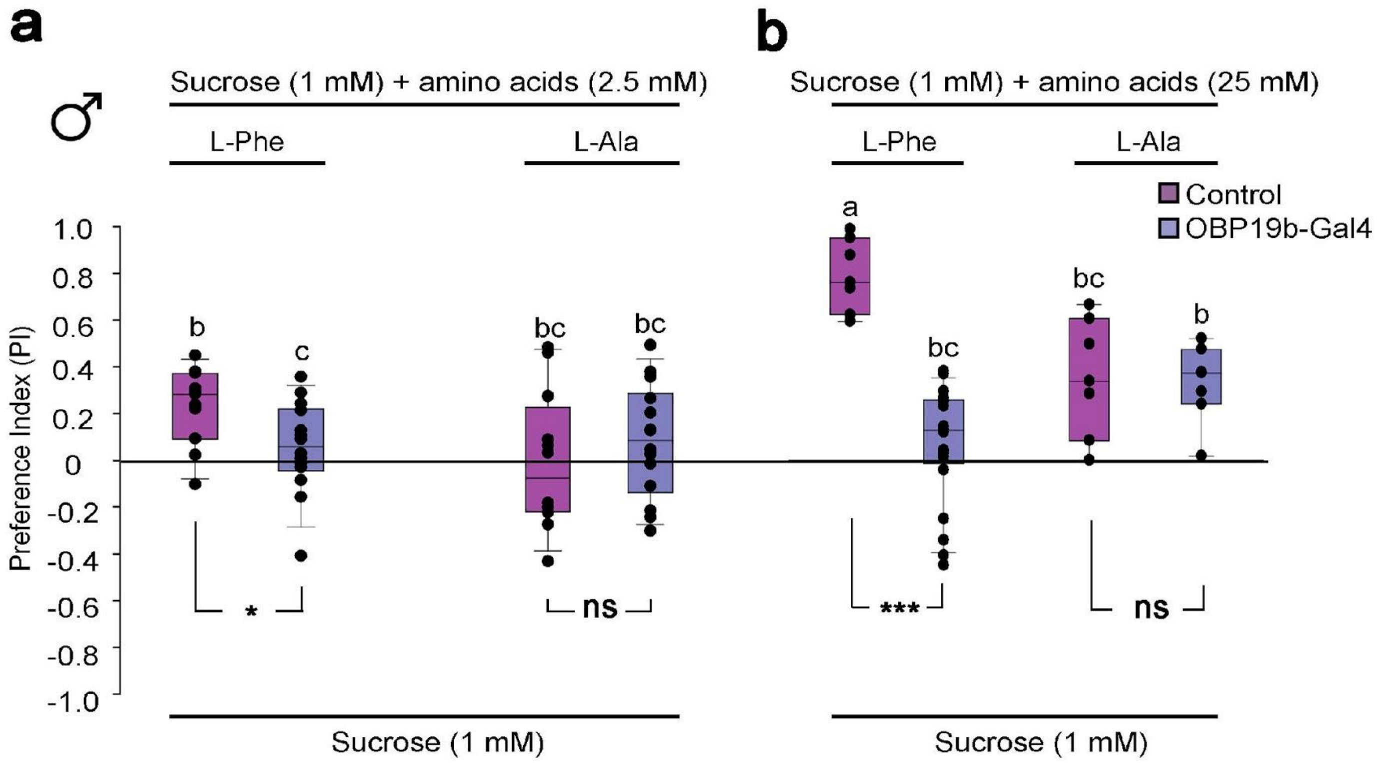

Supplementary Figure 4. Amino acid taste preference in males. (a) Preference for 2.5 mM L-phenylalanine (L-Phe) and L-alanine (L-Ala) was measured in control and OBP19b-Gal4 mutant flies. (b) Preference for 25 mM L-Phe and L-Ala in control and OBP19b-Gal4 mutant flies. Control and amino acid-rich solutions contained 1 mM sucrose. The amino acid solution either contained 2.5 or 25 mM of either amino acid. The letters indicate significant differences determined by the Kruskal Wallis test and post hoc Wilcoxon test: ns=nonsignificant; * p<0.05;** p<0.01; *** p<0.001). N=7-14.

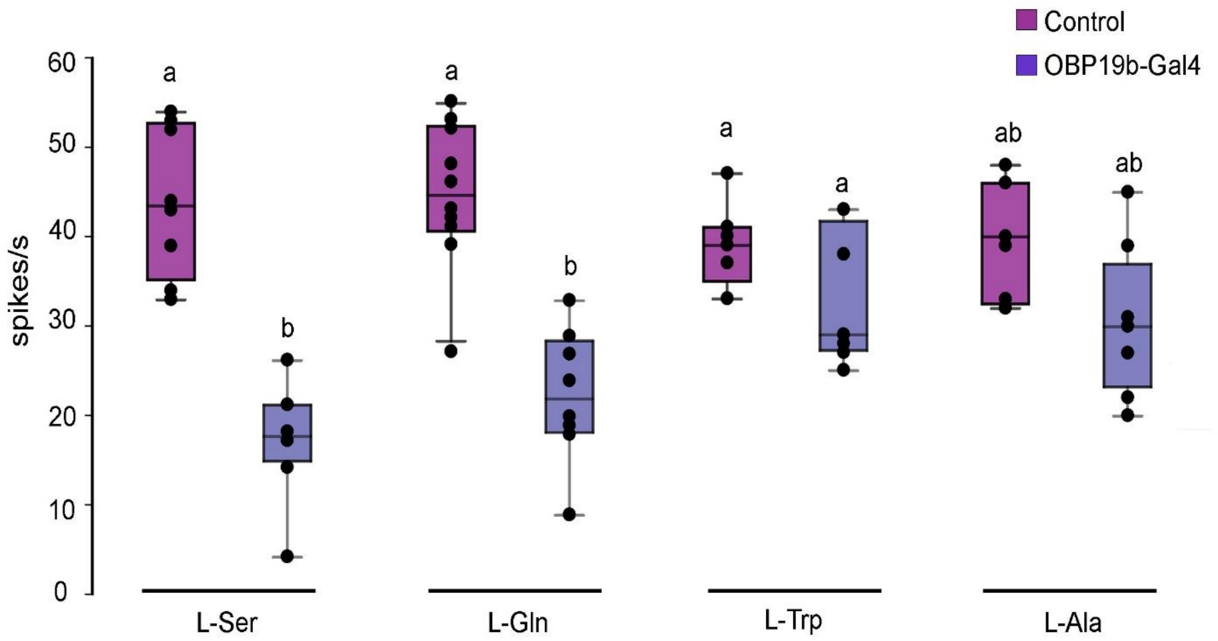

Supplementary Figure 5. Electrophysiological responses of s6 sensillum in control and mutant flies to several amino acids. The stimulus was contained in a glass microelectrode that capped the tip of the sensillum. Amino acids were dissolved in 1 mM KCl solution. Recordings were obtained from s6 sensilla in control CS and OBP19b-Gal4 mutant female flies. (a) Histograms represent the mean ± SEM for the number of spikes/s obtained in control and mutant flies in response to stimulation by 10 mM L-serine (L-Ser), L-glutamine (L-Gln), L-tryptophan (L-Trp) and L-alanine (L-Ala). Letters indicate significant differences determined with ANOVA and Tukey's post hoc test (a/b or b/c: $p<0.01$; a/c: $p<0.001$); N=10.

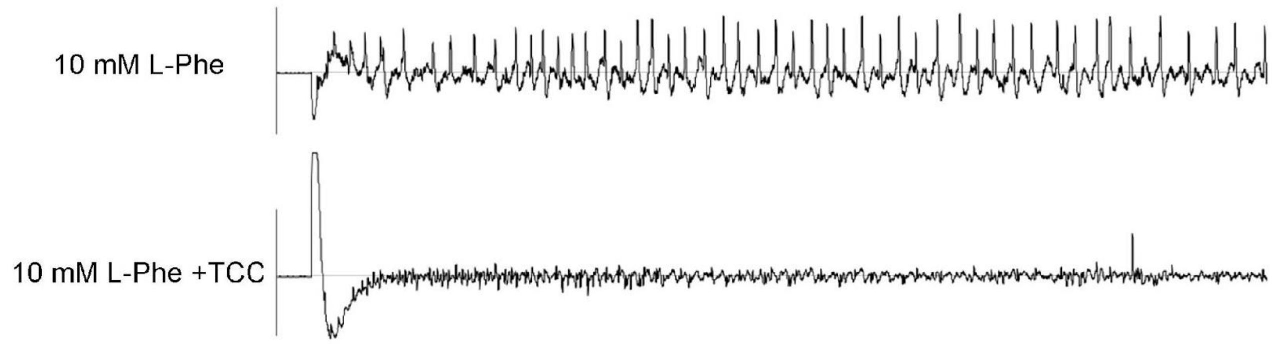

Supplementary Figure 6. Representative traces of tip recording data obtained from control flies after stimulation by 10 mM L-phenylalanine (L-Phe) with or without tricholine citrate (TCC).

a

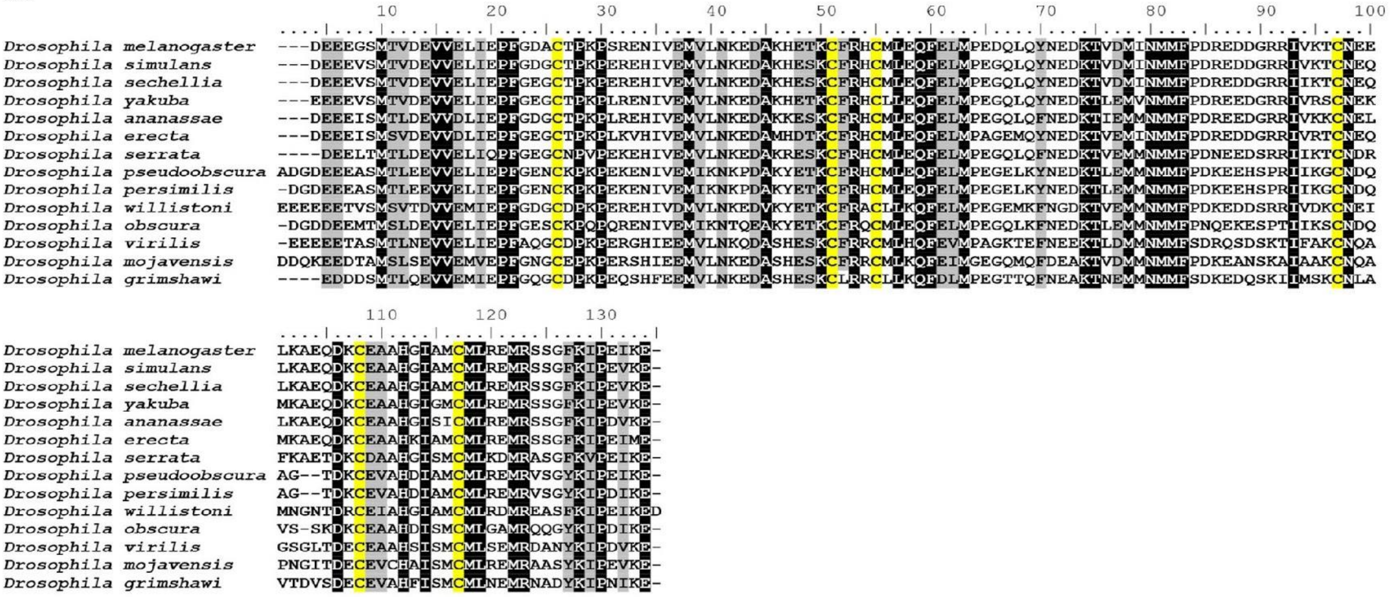

b

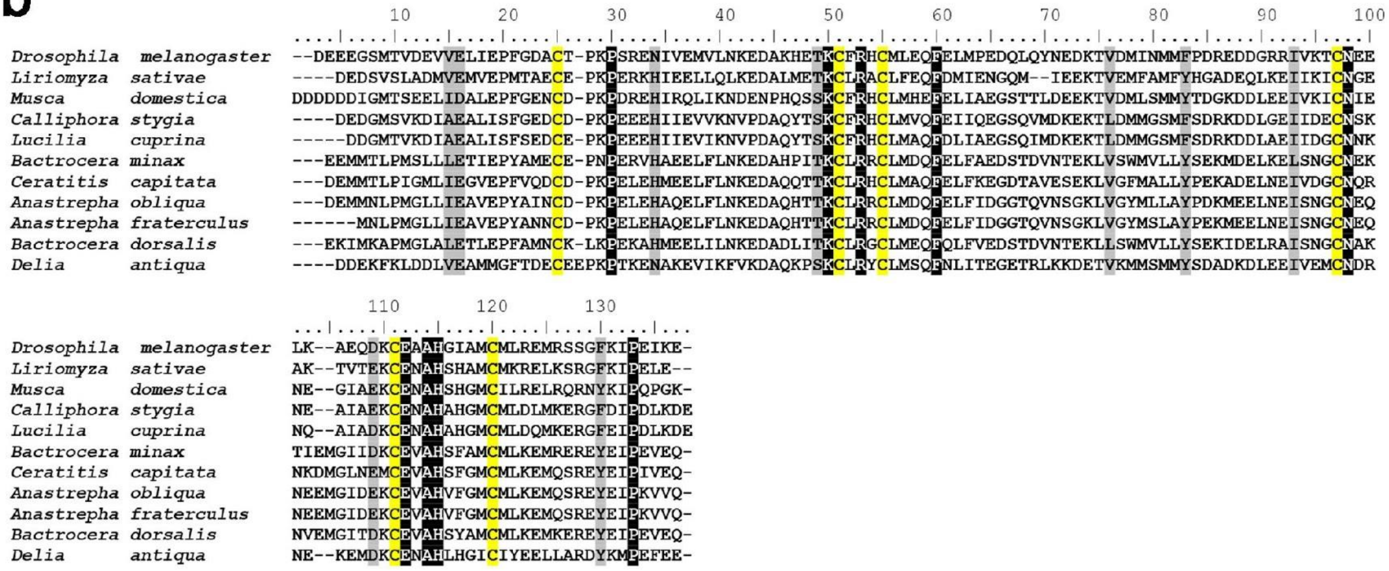

Supplementary Figure 7. OBP19b evolution and conservation. (**a**) Alignment of DmelOBP19b with the 13 OBP19b protein sequences of Drosophilidae identified in the currently available database. (**b**) Alignment of alignment of DmelOBP19b with the OBP19b sequences selected from non-Drosophiladea Diptera species. Conserved cysteines are highlighted in yellow, conserved and similar amino acid residues are shown in black- and grey- shaded background, respectively.

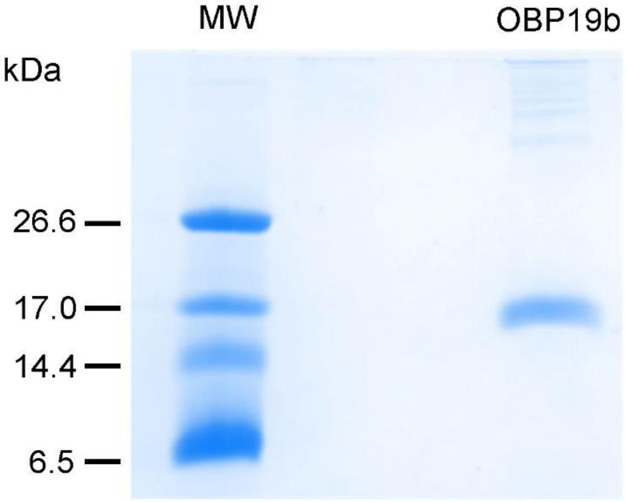

Supplementary Figure 8. SDS-PAGE analysis of purified OBP19b. Proteins were stained using Coomassie Brilliant Blue. The MW lane shows the molecular weight standard 6.5 – 26.6 kDa (Ultra Low Range marker, Sigma-Aldrich).

OBPs	% Identity
OBP57e	22.6
OBP57d	21.7
OBP57c	20.8
OBP19d	20.5
OBP56g	19.6
OBP19c	18.9
OBP56e	18.5
OBP57a	17.6
OBP56d	17.4
OBP57b	16.0
OBP56h	15.6
OBP49a	14.4
OBP56b	13.6
OBP83cd	10.8

Supplementary Table 1. Taste-associated OBP proteins of *D. melanogaster* represent a family of divergent proteins. Percentage identity matrix showing protein sequence alignment of DmelOBP19b with the 14 DmelOBPs expressed in adult gustatory appendages.
