# Supplementary material for: A conserved odorant binding protein is required for essential amino acid detection in Drosophila
Source: Commun Biol. 2019 Nov 22;2:425. doi: 10.1038/s42003-019-0673-2 (PMC6874667; doi:10.1038/s42003-019-0673-2)
Supplement: Supplementary file 2 — Description of additional supplementary files [file 42003_2019_673_MOESM2_ESM.docx]

**Description of additional supplementary files**

**Electrophysiology sheet:** The date of the recording, the fly strain and number, the recording number and the number of spikes are indicated in the tables. The concentration of the stimuli is indicated above each table.

**Legend:** control flies (CS); OBP19b mutant flies (OBP19b1).

**These data correspond to Figure 5 and Supplementary Figure 5**

**CAFE assay sheet:** The date of the experiment, the fly strain and sex, the preference index (pi) and the volume consumed of the solution containing the amino acid and the volume consumed of the solution without amino acid are indicated in the tables. The concentration of the amino acid tested is indicated above each table.

**Legend:** Volume consumed of the solution containing L-phenylalanine (Vcons L-phenyl); volume consumed of the solution containing L-ala (Vcons L-ala); volume consumed of the solution containing D-phenylalanine (V cons D-phe); volume consumed of the solution containing L-glutamine (Vcons L-glu); volume consumed of the solution with sucrose and without amino acid (Vcons sucrose); volume consumed of the solution without amino acid and sucrose (Vcons Buffer); control male flies (male CS); control female flies (female CS); OBP19b male mutant flies (male 19b); OBP19b female mutant flies (female 19b); rescue OBP19b flies (male rescue).

**These data correspond to Figure 4 and Supplementary Figures 3 and 4**

**Fluorescent binding assay sheet:**

**Concerning the screening of the fluorescent binding assay of OBP19b and 15mM amino acids or 15 mM tastants or 40 µM tastants (Figure 2a,b and c):** The ligand tested, the percentage of fluorescence (%Fluorescence) and the percentage of fluorescence displacement (%Fluorescence displacement) are indicated in the tables (column A, B and C). The ligand, the mean of percentage of fluorescence displacement (mean %Fluorescence displacement) and the SEM are indicated in tables (column F, G and H). The concentration of the tastants tested and the corresponding figures are indicated above each table.

**Concerning the binding curves data (Figure 2d, Supplementary Figure 2):** The concentration of the ligand, the mean of the percentage of fluorescence (%Fluorescence) of each ligand, and the SEM are indicated in the table (columns O-AZ; lines 1-12). The replicates of each tastant for each concentration tested are indicated in table (columns O-BJ; lines 22-118). The number and names of the amino acids tested are indicated in the **Number-names-AAs sheet.**
