# Supplementary material for: A conserved odorant binding protein is required for essential amino acid detection in Drosophila
Source: Commun Biol. 2019 Nov 22;2:425. doi: 10.1038/s42003-019-0673-2 (PMC6874667; doi:10.1038/s42003-019-0673-2)
Supplement: Supplementary file 4 — Reporting Summary [file 42003_2019_673_MOESM4_ESM.pdf]

nature research

COMMSBIO-19-0657-T
Corresponding author(s): Dr. Loic Briand, Dr. Jean-Francois Ferveur

Last updated by author(s): Oct 15, 2019

Reporting Summary

Nature Research wishes to improve the reproducibility of the work that we publish. This form provides structure for consistency and transparency in reporting. For further information on Nature Research policies, see Authors & Referees and the Editorial Policy Checklist.

Statistics

For all statistical analyses, confirm that the following items are present in the figure legend, table legend, main text, or Methods section.

n/a	Confirmed	
☐	☒	The exact sample size (*n*) for each experimental group/condition, given as a discrete number and unit of measurement
☐	☒	A statement on whether measurements were taken from distinct samples or whether the same sample was measured repeatedly
☐	☒	The statistical test(s) used AND whether they are one- or two-sided<br>*Only common tests should be described solely by name; describe more complex techniques in the Methods section.*
☒	☐	A description of all covariates tested
☐	☒	A description of any assumptions or corrections, such as tests of normality and adjustment for multiple comparisons
☐	☒	A full description of the statistical parameters including central tendency (e.g. means) or other basic estimates (e.g. regression coefficient) AND variation (e.g. standard deviation) or associated estimates of uncertainty (e.g. confidence intervals)
☒	☐	For null hypothesis testing, the test statistic (e.g. *F*, *t*, *r*) with confidence intervals, effect sizes, degrees of freedom and *P* value noted<br>*Give P values as exact values whenever suitable.*
☒	☐	For Bayesian analysis, information on the choice of priors and Markov chain Monte Carlo settings
☒	☐	For hierarchical and complex designs, identification of the appropriate level for tests and full reporting of outcomes
☒	☐	Estimates of effect sizes (e.g. Cohen's *d*, Pearson's *r*), indicating how they were calculated

Our web collection on statistics for biologists contains articles on many of the points above.

Software and code

Policy information about availability of computer code

Data collection	The data concerning the protein sequences were obtained using NCBI (BLASTP).<br>The rest of the data were experimental.
Data analysis	The sequence alignement were performed using CLUSTAL Omega alignment (https://www.ebi.ac.uk/Tools/msa/clustalo/).<br>The statistical analyses of binding, behavioral and electrophysiological assays were conducted using Excel xlstat version 19.6 and R version 3.5.1.

For manuscripts utilizing custom algorithms or software that are central to the research but not yet described in published literature, software must be made available to editors/reviewers. We strongly encourage code deposition in a community repository (e.g. GitHub). See the Nature Research guidelines for submitting code & software for further information.

Data

Policy information about availability of data

All manuscripts must include a data availability statement. This statement should provide the following information, where applicable:

- Accession codes, unique identifiers, or web links for publicly available datasets
- A list of figures that have associated raw data
- A description of any restrictions on data availability

All relevant data supporting the findings of this study are available from the corresponding author on request.

Field-specific reporting

Please select the one below that is the best fit for your research. If you are not sure, read the appropriate sections before making your selection.

☒ Life sciences ☐ Behavioural & social sciences ☐ Ecological, evolutionary & environmental sciences

For a reference copy of the document with all sections, see nature.com/documents/nr-reporting-summary-flat.pdf

Life sciences study design

All studies must disclose on these points even when the disclosure is negative.

Sample size	No sample size calculation was performed for binding assays, behavioral and electrophysiological responses. The sample size was chosen accordingly to statistical tests.
Data exclusions	No data were excluded.
Replication	For binding assays: the experiments were repeated 4 to 13 times.<br>For behavioral assay : for each Drosophila strain the experiments were repeated 7 to 15 times (each tube containing 10 flies).<br>For electrophysiological assay: for each Drosophila strain the experiments were repeated 8 to 12 times.
Randomization	Not relevant to our study.
Blinding	The investigators were blinded to group allocation during data collection and analysis.

Reporting for specific materials, systems and methods

We require information from authors about some types of materials, experimental systems and methods used in many studies. Here, indicate whether each material, system or method listed is relevant to your study. If you are not sure if a list item applies to your research, read the appropriate section before selecting a response.

Materials & experimental systems

n/a	Involved in the study	
☒	☐	Antibodies
☒	☐	Eukaryotic cell lines
☒	☐	Palaeontology
☐	☒	Animals and other organisms
☒	☐	Human research participants
☒	☐	Clinical data

Methods

n/a	Involved in the study	
☒	☐	ChIP-seq
☒	☐	Flow cytometry
☒	☐	MRI-based neuroimaging

Animals and other organisms

Policy information about studies involving animals; ARRIVE guidelines recommended for reporting animal research

Laboratory animals	Drosophila melanogaster, strains: Obp19b1-null mutant, ASE5-GFP, UAS-OBP19b, nompA-GAL4, Canton-Special (CS), sex: male and female, age: 7 days old, 10 flies for CAFE assay; 2–3-day-old flies for electrophysiology.
Wild animals	The study did not involve wild animals.
Field-collected samples	The study did not involve samples collected from the field.
Ethics oversight	No ethical approval or guidance was required, studies on Drosophila does not need ethical approval.

Note that full information on the approval of the study protocol must also be provided in the manuscript.
